# Strength and Microstructural Characteristics of Fly Ash–Waste Glass Powder Ternary Blended Concrete

**DOI:** 10.3390/ma18194483

**Published:** 2025-09-25

**Authors:** Moruf O. Yusuf, Khaled A. Alawi Al-Sodani, Adeshina A. Adewumi, Muyideen Abdulkareem, Ali H. Alateah

**Affiliations:** 1 Department of Civil Engineering, College of Engineering, University of Hafr Al Batin, P.O. Box 1803, Hafr Al Batin 39524, Saudi Arabia; kalsodani@uhb.edu.sa (K.A.A.A.-S.); adeshina@uhb.edu.sa (A.A.A.); ali.alateah@uhb.edu.sa (A.H.A.); 2 Department of Civil Engineering & Quantity Surveying, Military Technological College, Muscat 111, Oman; muyikareem@gmail.com

**Keywords:** glass waste, admixtures, microstructure, construction materials, fly ash, high-strength concrete

## Abstract

To reduce the proliferation of greenhouse gases in the construction industry, ternary blended concrete comprising fly ash (FA) powder, waste glass (WG) powder, and ordinary Portland cement (OPC) was developed such that the WG to total binder varied from 0 to 20% at intervals of 5% (C_80_FA_20-x_WG_x_:x = WG/(WG + FA + OPC)). The developed concrete was investigated for water absorption, workability, 28-day compressive strength, binder phases, bond characteristics, microstructure, and elemental composition of the concrete. The mixture proportions of C_80_FA_15_WG_5_ and C_80_FA_10_WG_10_ exhibited better consistency and water absorption than the OPC concrete (C_100_FA_0_WG_0_). Furthermore, the 28 d strength of C_80_FA_15_WG_5_ marginally outperformed those of C_80_FA_10_WG_10_ and C_80_FA_20_WG_0_. The sample with equal proportions of FA and WG (C_80_FA_10_G_10_) was more amorphous owing to the disappearance of the hedenbergite phase (CaFeSi_2_O_6_) and conversion of tobermorite (CSH) to C-A-S-H. C_80_FA_10_WG_10_ also exhibited better microstructural stability than FA + OPC concrete (C_80_FA_20_G_0_), owing to the pore-filling of the microcracks within the matrix. Finally, higher Si/Ca, Ca/Al, and Si/Al ratios were recorded in C_80_FA_10_WG_10_ than in the case of FA preponderating WG in ternary blending. Finally, structural concrete can be produced through the ternary blending of glass waste, fly ash, and OPC, thereby promoting the valorization of solid waste and a sustainable environment.

## 1. Introduction

The proliferation of greenhouse gases is a significant challenge that requires attention to effectively address the problem of global warming. Cement production industries contribute significantly to the proliferation of carbon dioxide during the calcination of limestone in the cement manufacturing process. Solid waste emanates from different sources, such as agro, cement, and power generation industries. The indiscriminate deposition of solid waste in landfills constitutes environmental pollutants that can negatively affect public health. Many materials are used as supplementary materials in normal- and high-performance concrete to achieve economic and environmental waste reduction in concrete production [1,2]. These solid waste materials include silica fume (SF), fly ash (FA), metakaolin (MK), and steel slag. In recent times, stone dust, palm oil fuel ash (POFA), pulp and waste paper, and waste glass (WG) have also been used to achieve better strength and durability performances in concrete and mortar [3,4]. WG constitutes 7% of the world’s solid waste and 4.2% of the 12.3 million tons of the total municipal solid waste (MSW) generated in the US in 2018 [5]. The percentage of MSW deposited in landfills amounted to 5.2% (7.6 million tons) (United States Environmental Protection Agency) [6]. The annual amount of recyclable glass in Australia is about one million tons; approximately 50% was reported as combusted glass [7]. Effective preservation and efficient WG management processes, as well as a reduction in annual ordinary Portland cement (OPC) consumption, are essential to reducing greenhouse gases and accumulating solid waste in the environment.

Several researchers have studied the contributions of WG to the mechanical and durable properties of concrete. This paves the way for the utilization of WG in fragmented and powdered forms for fine aggregates and supplementary cementitious additive applications, respectively. This effort reduces environmental pollution, which could negatively affect public health owing to its accumulation in the landfill [8,9,10,11]. Other types of glass, such as cathode ray tubes, solar panels, and fluorescent lamps, have also been used to produce mortar and concrete. Replacing 20% ordinary Portland cement (OPC) with WG powder contributes to the 90-day strength of concrete [12]. Tan et al. also reported the contribution of WG to the density and rheology of concrete when used as fine aggregate, while Lu et al. [13] linked the extension of the setting time, hydration process, and flowability to the content of WG powder in concrete. Rahma et al. [14] also reported a reduction in the workability of glass-blended concrete. Elsewhere, hollow glass microsphere waste has been used as an anchor for polymer and lightweight mortar production [15].

Furthermore, the size and color of glass can control its chemical reactivity [16,17]. The inclusion of WG reduces early strength due to the dilution effect, which prevents the hydration of alite during the early days of strength development [7]. WG particles also contribute to durability and transport properties together with microstructural refinement by retaining non-evaporable water within the capillary pores [18,19]. Kawalu et al. [20] used fragmented WG as fine aggregates in geopolymer and OPC mortar synthesis. They discovered that a reduction in drying shrinkage compromised the strength of the binder. Althoey et al. [21] replaced fine aggregate with WG and fiber in natural fiber-reinforced concrete to achieve eco-friendly concrete. Tan et al. [10] investigated the contribution of milled WG as sand in mortar and asserted that less than 30% of the additive could contribute to electrical resistivity and absorption. Furthermore, Yusuf et al. [22] explored the performance of WG in synergy with silica fume (SF) and OPC in ternary blending for a deeper understanding of its contribution to fresh, hardened, and microstructural properties.

Fly ash powder (FA), on the other hand, is a by-product of the combustion of coal in power-generating stations. It has been reported that approximately 30.1 million tons of fly ash was produced in the US, while only 42% was put into beneficial use. This promotes its utilization in concrete production as a pozzolanic material or geopolymer precursor. The silica and alumina contents of FA have made it more relevant because of its contribution to the secondary hydration process in the OPC binder or aluminosilicate precursor in geopolymer synthesis [23]. Its higher alumino silicate (SiO_2_ and Al_2_O_3_) and lower lime (CaO) contents relative to WG and OPC could make its combination with WG more beneficial for ternary binder synthesis [24]. Several studies have reported that the addition of FA contribution to concrete enhances workability and mechanical properties; likewise, there is a decrease in permeability and better sulfate resistance [3].

For instance, FA and mine tailings (MT) have been reported to enhance concrete consistency without loss in flexural and split tensile strength [25]. Moreover, Goksen et al. [26] studied the performance of WG and FA separately as partial supplementary cementitious materials (SCMs) for OPC to mitigate the effect of alkaline silica reactivity (ASR) through a reduction in concrete porosity and water absorption while increasing the binder strength. Jurczak et al. [27] reported that the replacement of WG blended concrete outperformed FA blended concrete in terms of strength and durability. FA and WG have also been used for glass production with a significant increase in mechanical and surface properties [28,29]. Sunarsih et al. [30] also used FA to improve the flexural and sorptivity properties in concrete. Be that as it may, there could be some benefits in the combination of fly ash and WG due to the potency to improve the properties of OPC-based concrete due to the high silica and alumina in both materials compared to OPC. The use of WG with FA could also reduce the effect of the mercury content of the FA, which has been reported to be 1 ppm, through chemical interaction and the dilution effect [31,32,33].

Finally, despite the plethora of studies on the use of WG and FA as additives in concrete production, there has not been a detailed study on the ternary blending of WG, FA, and OPC together with a deeper understanding of the microstructural characteristics and elemental composition of such synthesis. Therefore, this study investigated the performance of ternary blended concrete, including OPC, WG, and FA, in terms of water absorption, workability, and compressive strength. The developed concrete consisted of OPC (80–100%) and WG + FA of 20%, such that WG varied with FA in different ratios. Therefore, this study seeks to provide a deeper understanding of the transport, strength, and microstructural characteristics of ternary blended concrete at different ages for structural applications. Workability, water absorption, compressive strength, and microstructural analyses were performed using scanning electron microscopy with energy-dispersive spectroscopy (SEM/EDS), X-ray diffraction (XRD), and Fourier transform infrared (FTIR) spectroscopy techniques. Finally, it is expected that this study will contribute to and promote the use of WG+FA-based ternary concrete for sustainable infrastructural development in regions where these two materials contribute significantly to environmental challenges, such as solid waste accumulation in landfills, which could pose a threat to public health.

## 2. Materials and Methods

### 2.1. Binder Raw Materials

**Figure 1:**
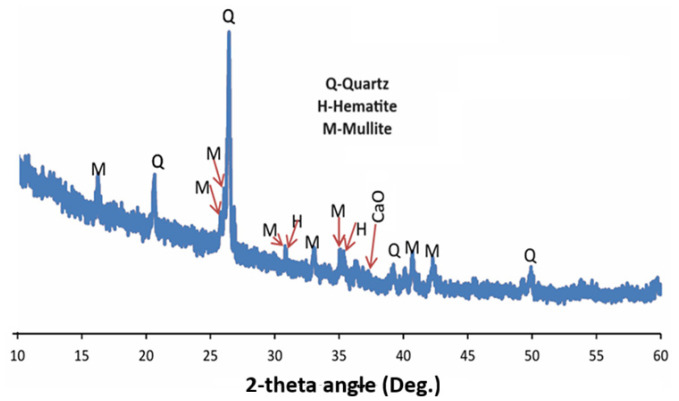
XRD diffractogram of Class F fly ash.

**Figure 2:**
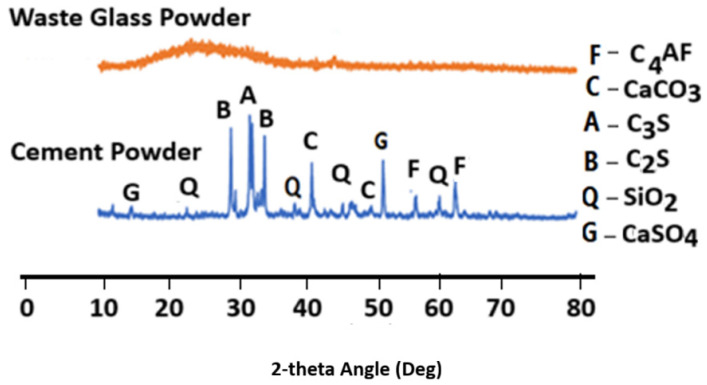
XRD diffractogram of the glass waste and cement powder.

**Figure 3:**
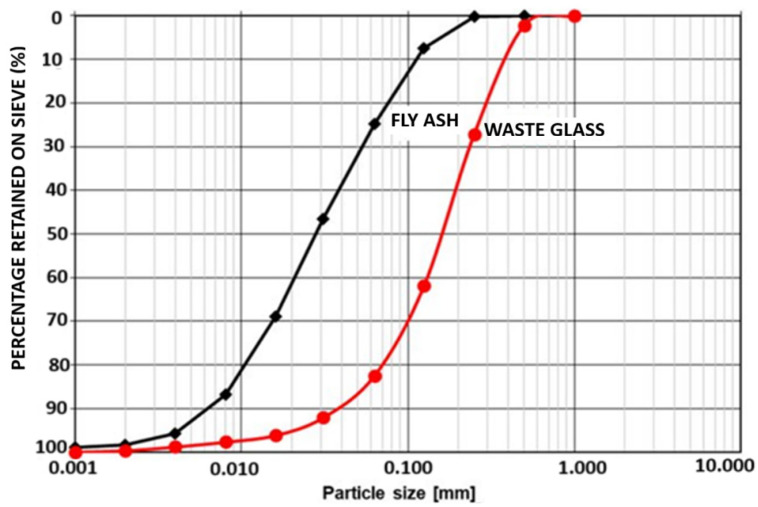
Particle size analysis of the fly ash and glass waste powders.

Ordinary Portland cement (OPC) Type 1 was used in accordance with ASTM C 150, [34] with an apparent specific gravity of 3.15. Fly ash (FA) was supplied by High-tech Fly Ash (India) Private Limited with the oxide composition determined by Rigaku X-ray fluorescence (XRF), Tokyo, Japan. The results of XRF and XRD of flyash are as shown in Table 1, while the X-ray diffractogram (XRD) of waste glass, flyash and cement are as shown in Figure 1 and Figure 2. Figure 3 shows the particle size analysis of both fly ash and waste glass powders determined using the time of transition method with an Ankersmid CIS-50, Nijverdal, Netherlands particle analyzer.

Waste glass powder (WG) was obtained from a dumpsite along Sinaya Road, Hafr Al-Batin, Kingdom of Saudi Arabia. The WG was placed and crushed in a Los Angeles grinding machine and then placed in an oven at 105 °C for moisture removal. It was then ground with an electronic grinder with a titanium blade, a power rating of 1.4 kW, and a voltage and frequency of 220 V and 60 Hz, respectively.

### 2.2. Aggregates

Natural dune sand passing a 2.36 mm sieve (No. 8) and complying with ASTM C 33 [35] was used as fine aggregates. Its fineness modulus was 3.3, and the relative density (water) was 2.71. The coarse aggregate was limestone minerals with sizes ranging from 10 to 20 mm, which was used under saturated surface dry (SSD) conditions (Table 2). The specific gravities of all the materials are listed in Table 3.

### 2.3. Superplasticizer

Glenium^®^ superplasticizer of 0.5 wt.% of binders (cement, fly ash, and glass powder) was mixed with water to enhance the workability of the concrete. Glenium has been reported to have a better performance and longer retention of slump than concrete mixtures containing melamine, naphthalene, and high-range polycarboxylate water-reducing admixture.

### 2.4. Mix Design and Sample Preparations

#### 2.4.1. Mix Design

Concrete was produced by maintaining a water-to-binder ratio and fine-to-total aggregate ratio of 0.4 (Table 4). The density of the concrete was approximately 2400 kg/m^3^. The total binder, consisting of OPC, FA, and WG, was 350 kg/m^3^. In the ternary blended concrete, the combined percentage of WG and FA was 20 wt.% (70 kg), while OPC accounted for 80 wt.% (280 kg). For the control sample, OPC was at 100%, with FA and WG at 0%. The waste glass (WG/(FA + WG + OPC)) and fly ash (FA/(FA + WG + OPC)) contents in the ternary blended samples were varied as 0%, 5%, 10%, 15%, and 20%.

#### 2.4.2. Sample Preparations

The sample was prepared according to the mix design presented in Table 4 by first adding 75% of the total water mixed with superplasticizers in the mixer. The OPC, WG, and FA powders were then placed in a rotary mixer and mixed for 3 min. Fine and coarse aggregates were then added and mixed for an additional 4 min, and the remaining water (25%) was subsequently added. The total mixture was thoroughly mixed homogenously for an additional 4 min before being emptied into the oil-smeared mold of 100 × 100 × 100 mm after being properly compacted into three layers. The surface of the sample in the mold was smoothed by a hand trowel and then covered with a polythene sheet to prevent moisture loss. The samples were demolded and kept inside a curing tank at room temperature (25 °C) in the laboratory until they were ready for testing after allowing them to drain for at least 6 h. Subsequently, the slump was determined using a cone test (ASTM C143) with an oil-smeared mold of 100 × 200 × 300 mm.

### 2.5. Sample Designation

The mixture proportions of cement (OPC), waste glass (WG), and fly ash (FA) in different proportions are presented in Table 4. The percentage of OPC was maintained at 80 wt.% and 100% when both FA and WG were made to be 20%. The samples were categorized into three groups: C_100_FA_0_WG_0_ (OPC concrete), C_80_FA_20_G_0_ (FA binary blended binder), and C_80_FA_20-x_G_x_ (ternary blended concrete, x = 5, 10, 15, and 20%).

### 2.6. Experimental Testing Method

#### 2.6.1. Workability

The workability of the concrete was tested using a slump cone test in accordance with ASTM C 143 [37]. The difference in the height of the concrete and the cone measured by the meter rule was recorded as the slump value (in mm).

#### 2.6.2. Water Absorption

The 28-day cubic concrete samples (100 mm × 100 mm × 100 mm) of known mass were submerged in water for 24 h and thereafter mopped with a towel to determine their saturated surface dry mass, M_ssd_. The samples were then dried in an oven (105 °C, 24 h). The water absorption (at 28 days) was calculated using Equation (1):

(1) $$\mathrm{Water}\mathrm{absorption}=\frac{M_{ssd}-M_{oven}}{M_{oven}}\times 100$$

#### 2.6.3. Compressive Strength Test

The compressive strengths of the ternary blended concrete (C_80_FA_20-x_WG_x_) at 7, 14, and 28 days were determined using cubic sample sizes of 100 × 100 × 100 mm in accordance with BS EN 12390-3 [38]. The samples were crushed using a universal testing machine at a loading rate of 0.9 kN/s. The average of the three samples was recorded to determine the desired strength on specific days.

#### 2.6.4. Characterization and Morphology of the Specimens

Scanning electron microscopy/energy-dispersive X-ray spectroscopy (SEM + EDS), for microstructural analysis of the samples was obtained by using Japan, JEOL instrument model 5800 L Japan (JEOL, Tokyo, Japan) with an accelerating voltage of 20 kV model 5800 LV. The sample used was a fragmented 28-day solid paste specimen was fastened onto the holder using carbon tape and then sputter-coated with a thin gold lining to prevent surface charging and improve the quality of the image. Fourier transform spectroscopy (FTIR) instrument manufactured Perkin Elmer 880 spectrometer (Perkin Elmer, Springfield, IL, USA), China was used to determine the bond characteristics of the 28-day pulverized sample passing through 75-microns sieve before being blended with KBr at 1:9 by volume. Further, XRD Bruker instrument (Bruker, Billerica, MA, USA), model d2-Phaser manufactured from Germany was used to determine the nature of hydration products after 28 days. The equipment radiation was Cu Ka radiation (40 kV, 40 mA) working by continuous scanning within a 2-theta angle range of 4–80° at a scan speed of 2.5°/min. To prepare samples for XRD, 28-day paste hydrated sample was ground and then passed through a 75-micron sieve, evenly packed, spread and placed in the instrument sample holders.

## 3. Discussion of Results

### 3.1. Workability of Glass–Fly Ash Ternary Blended Concrete

**Figure 4:**
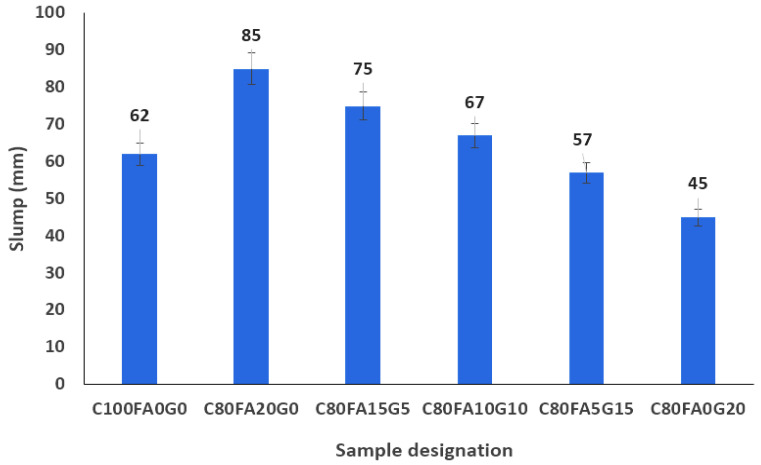
Workability of fly ash–waste glass ternary blended concrete.

Figure 4 shows that fly ash (FA)-blended concrete (C_80_FA_20_WG_0_) had the highest consistency of all the mixtures owing to its spherical particles. This also corroborates the finding that FA enhanced the consistency of a concrete mixture through the lubricating interparticle interactions. The flowability of C_80_FA_20_WG_0_ was 37.1% higher than that of OPC concrete (C_100_FA_0_WG_0_). By incorporating the combined WG and FA in varying proportions of 5–10% in partial replacement for OPC (C_80_FA_15_WG_5_ and C_80_FA_10_WG_10_), the workability was reduced by 21% and 8.1%, respectively, when compared with C80FA20WG0. In comparison with OPC concrete (C_100_FA_0_WG_0_), the consistencies of C_80_FA_15_WG_5_ and C_80_FA_0_G_20_ were reduced by 8.1% and 27.4%. The use of 5% WG and 15% FA (C_80_FA_15_WG_5_) could attain a slump value of 75 mm, which is sufficient for fresh concrete for many structural applications, as shown in Figure 3, where the workability of pure OPC (C_100_FA_0_WG_0_) concrete is 62 mm. Furthermore, the workability of C_80_FA_20_WG_0_ reduced by 11.8%, 21.2%, 32.9%, and 47.10%, respectively, upon adding 5, 10, 15, and 20% of WG as x values in C_80_FA_20-x_WG_x_, respectively. The synergy of both WG and FA impeded the lubricating effect of FA particle interactions with the aggregates, thereby increasing the energy required to overcome interparticle friction. The reduction in consistency was due to the irregular shapes of WG, thereby enhancing the interparticle friction that hinders the flow. Thus, the impact of WG on FA-blended concrete in affecting the workability performance of the ternary blended concrete could vary depending on the WG/FA ratios. This provides the opportunity for economic use of waste materials to enhance and modify the fresh properties of concrete, working towards achieving a desired concrete workability.

### 3.2. Absorption of Fly Ash–Waste Glass Ternary Blended Concrete

**Figure 5:**
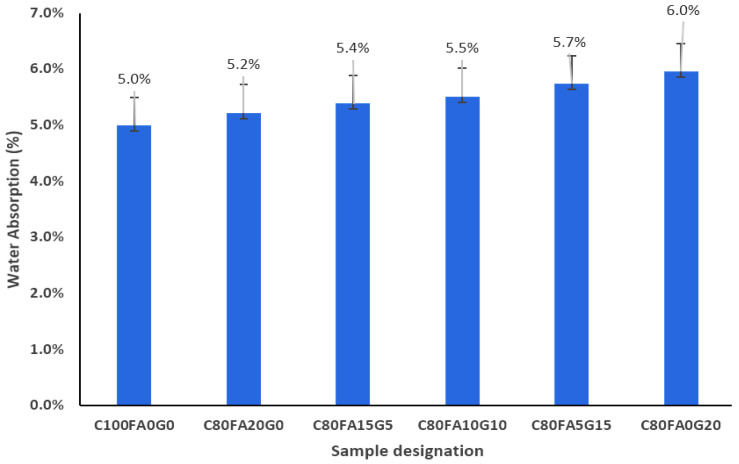
Water absorption in fly ash–glass ternary blended concrete.

Figure 5 shows that incorporation of 20% of WG+FA in varying quantities (WG/(WG + FA + OPC) = 0%, 5%, 10%, 15%, and 20%) into the 28 day ternary blended concrete (C_80_FA_20-x_WG_x_) linearly increases the water intake capacity. The presence of only FA merely increased the absorption of OPC concrete by 4%. Similarly, Golewski [39] reported a 6% increase in absorption of OPC concrete upon incorporating 20% FA.

This implies that the absorption of ternary concrete increases with the preponderance of WG over FA because of its irregular particle shape, larger sizes (D_10_, D_50_), and greater capillary action, which favors water permeability in the interfacial transition zone between the aggregates, particles, and paste matrix. In addition, a high WG/FA ratio could be responsible for the widening of capillary pores, thereby decreasing the tortuosity of the binder matrix. Figure 5 shows that the percentages of water intake obtained in C_80_FA_20_WG_0_, C_80_FA_15_G_5_, C_80_FA_10_WG_10_, C_80_FA_5_WG_15_, and C_80_FA_0_WG_20_ increased by 4, 8, 10, 14, and 20%, respectively, in comparison with the OPC concrete (C_100_FA_0_WG_0_). This is consistent with previous reports from Guo et al. [32]. This indicates that if there is more WG than FA in concrete production, it will enhance the porosity of concrete as the absorption increases with the WG content in the samples.

### 3.3. Compressive Strength of Glass–Fly Ash Ternary Blended Concrete

**Figure 6:**
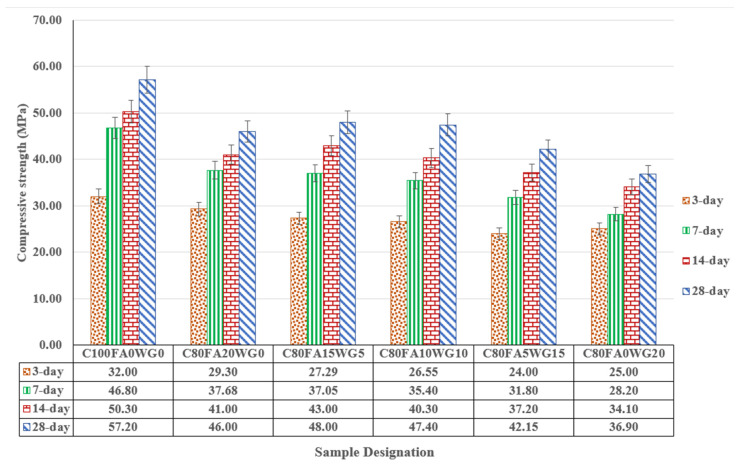
Compressive strength of glass–fly ash ternary blended concrete.

The compressive strength of ternary blended concrete (C_80_FA_20-x_WG_x_) shown in Figure 6 indicates that 3-d early strength of C_80_FA_20-x_WG _x_ decreases with WF/FA ratios. The lowest 3-d early strength C_80_FA_0_G_20_ and C_80_FA_20_WG _0_ were 25 MPa and 29.3 MPa, respectively while it was 32 MPa in OPC concrete (C_100_FA_0_WG_0_). 

Furthermore, the 3-day strengths recorded in C_80_FA_20_ WG_0_, C_80_FA_15_WG_5_, C_80_FA_10_WG_10,_ C_80_FA_5_WG_15_ and C_80_FA_0_WG_20_ reduced by 8.43, 14.71, 17.03, 25, and 21.88%, respectively in comparison with OPC concrete. The reason for the decrease could be adduced to early day dilution effect on strength development as induced by FA and WG. Moreover, the compressive strength was noticeably decreasing with increase in WG/FA ratio with the lowest value recorded in WG blended sample (C_80_FA_0_WG_20_). It can be said that adding FA (10–15%) in synergy with WG enhanced the strength of the ternary blended concrete better than what is obtainable in binary blended concrete of OPC-FA or OPC-WG-based independently. Thus, higher WG/FA ratios had more debilitating impacts than the lower WG/FA ratio on the achievable strength in the synthesis ternary blended concrete. 

**Figure 7:**
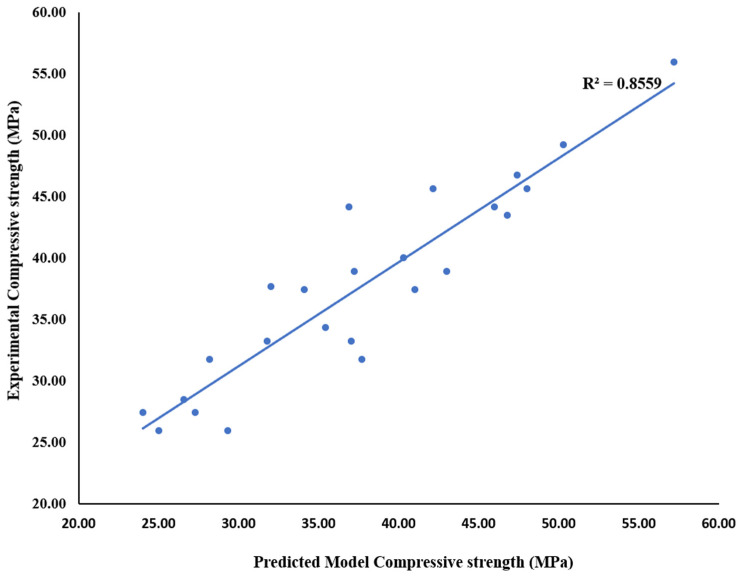
Prediction of strengths in (WG + FA + OPC) ternary concrete using regression model.

A similar trend was also observed at 7–14 day of strength development when the strength recorded in OPC concrete (C_100_FA_0_WG_0_) is compared to those recorded in blended samples. For instance, C_80_FA_20_WG_0_ C_80_FA_15_WG_5_ C_80_FA_10_WG_10_, C_80_FA_5_WG_15_ and C_80_FA_0_WG_20_ are lower than the result obtained in OPC concrete by educed by 8.9, 16.1, 13.8, 17.0 and 21%, respectively. The optimal strength of 46–48 MPa could be achieved in ternary blending structural concrete (C_80_FA_20-x_WG_x_) with adequate mixing, curing and compaction when x varied between 0, 5, and 10% while WG/FA ratios were within 0.33–1.0. Figure 7 depicts the relationship between the experimental and predicted strength models in WG-FA ternary blended concrete with high coefficient of determination of 0.86 (R^2^ = 0.856) with correlation coefficient of 0.93 as shown in Equation (2). The parameters used in the model include age (3,7,14, and 28 days), OPC (80–100%), WG (0–20%) and FA (0–20%) together with their possible combinations.

(2) $${f^{\prime }}_{c}=19.95Age^{0.2387}+3.21\times 10^{-217}{\left(OPC\right)}^{85.52}+{2.883\times 10}^{-6}{\left(FA\times WG\right)}^{1.9277}\;+9.63\times 10^{-50}{\left(FA\times OPC\times WG\right)}^{9.0085}$$

### 3.4. Microstructural Characteristics and Elemental Analyses of the Products

**Figure 8:**
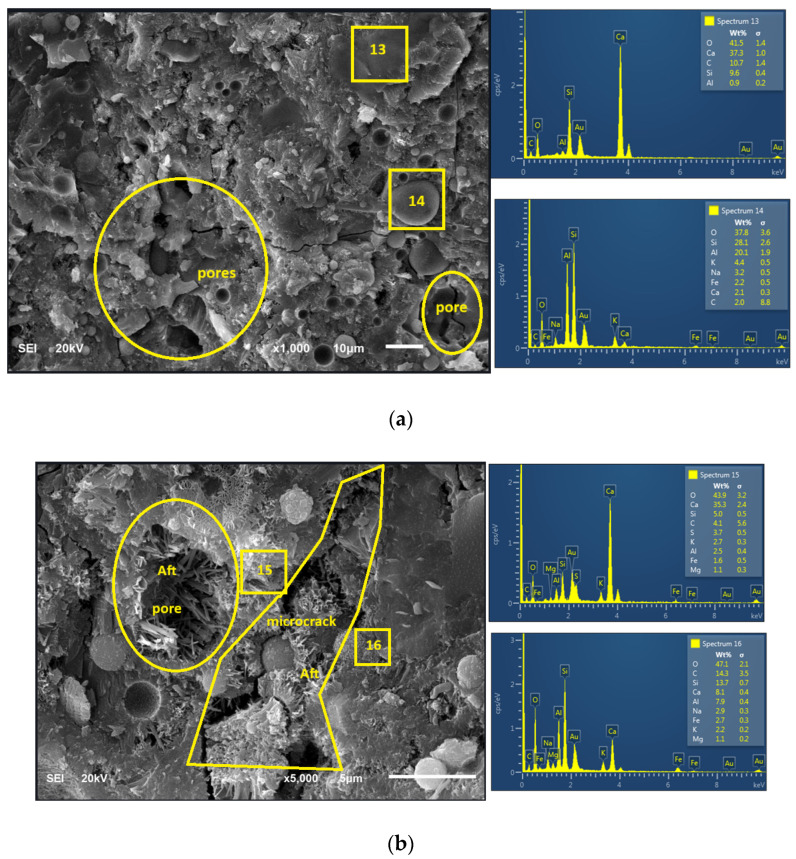
Morphology of 28-day fly ash + OPC cement binary blended paste (C_80_FA_20_WG_0_) at different Magnifications and resolutions (**a**) Mag. X1000 (10 microns) (**b**) Mag. X5000 (5 microns).

**Figure 9:**
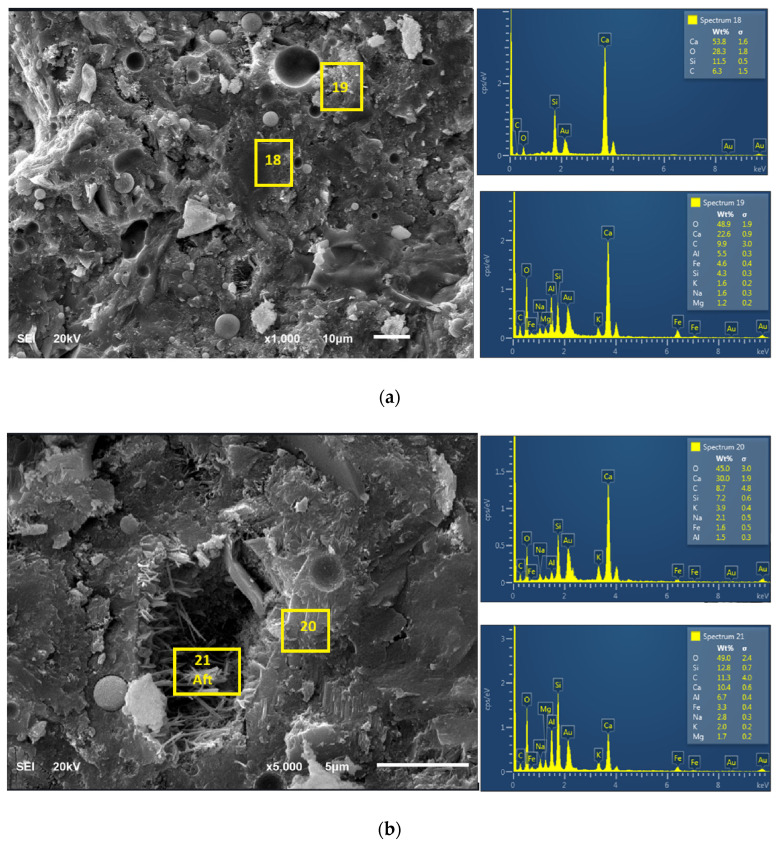
Morphology of ternary blended WG + FA + OPC (C_80_FA_10_WG_10_) paste at different Magnifications and resolutions (**a**) Mag. X1000 (10 microns) (**b**) Mag. X5000 (5 microns).

Figure 8 and Figure 9 show the morphology of 28-day paste of FA-OPC binary blended paste (C_80_FA_20_G_0_) and OPC + FA + WG ternary blended paste (C_80_FA_10_WG_10_). The microstructure of C_80_FA_20_G_0_ is characterized by discontinuity and microstructural weakness due to microcracks and pores while that of C_80_FA_10_WG_10_ gives a better microstructural stability (Figure 9). This shows that WG provided a pore-filling or packing effect in the presence of FA within the OPC capillary pores. EDS reveals that Ca/Si is reduced from 3.82 (C_80_FA_20_WG_0_) to 3.36 in C_80_FA_10_G_10_ due to the presence of high Si/Ca, as noted in the XRF results and EDS in Table 1 and Table 5, respectively. This suggests that silicate re-organization could be better achieved in a WG + FA + OPC ternary system (C_80_FA_10_WG_10_) in comparison with binary FA+OPC systems (C_80_FA_20_WG_0_). Furthermore, C_80_FA_10_WG_10_ (Figure 9) has a smoother and denser morphology with a lower pore distribution compared to C_80_FA_20_WG_0_ (Figure 8a).

**Figure 10:**
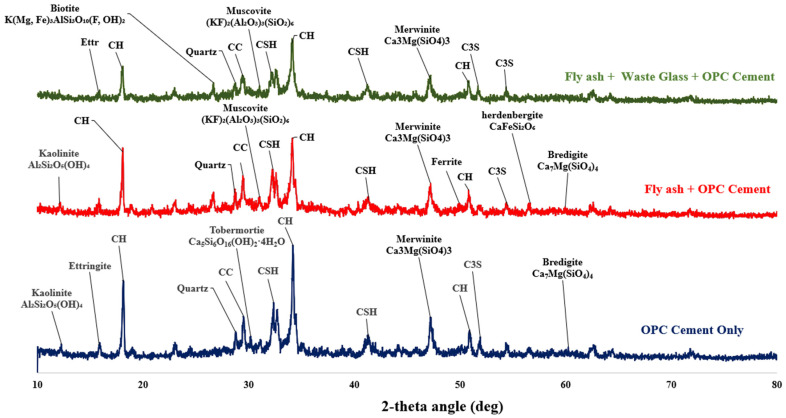
XRD diffractogram of fly ash–glass blended binder (C_80_FA_10_WG_10_—top), fly ash blended binder (C_80_FA_20_WG_10_—middle), and OPC cement binder (C_100_FA_0_WG_0_—bottom).

The weakness in the microstructure of C_80_FA_20_WG_0_ is because of the dilution effect and the formation of C-A-S-H within C-S-H due to the presence of more Al in FA. The presence of sulfate in OPC reacts with alumina to form Aft (ettringite). The pore distribution across different regions in the microstructure can be clearly seen in Figure 8, whereas this is localized and concentrated in Figure 9. This indicates that the presence of WG in the ternary binder could help to localize Aft (ettringite) formation [40]. The XRD diffractogram in Figure 10 indicates that ettringite in C_80_FA_10_WG_10_ was less crystalline compared to that in the C_80_FA_20_WG_0_ and C_100_FA_0_WG_0_ systems. The tobermorite (C-S-H) phase was also more visible in the OPC binder (C_80_FA_0_WG_0_) but became amorphous in C_80_FA_20_WG_0_ and C_80_FA_10_WG_10_.

In addition, from the C_80_FA_20_WG_0_ system (Figure 8), Si/Al was 3.0 and became 3.69 in C_80_FA_10_WG_10_. Further, a low Si/Al ratio could lead to the formation of calcium aluminosilicate hydrate (C-A-S-H) instead of calcium silicate hydrate (CSH) [41]. Table 5 also points to more Ca/Al (12.5) in ternary blending (C_80_FA_10_G_10_) compared to a binary binder (C_80_FA_20_WG_0_), whose value is 11.46.

### 3.5. X-Ray Diffraction of the Fly Ash–Glass Ternary Blended Binder

Figure 10 shows the prominent features common to all three binders, which include ettringite (Ca_6_Al_2_(SO_4_)_3_(OH)_12_·26H_2_O), calcium silicate hydrate (C-S-H) (Ca_5_Si_6_O_16_(OH)_2_.4H_2_O), calcite (CaCO_3_), merwinite (Ca_3_Mg(SiO_4_)_3_), quartz (SiO_2_), and portlandite (Ca(OH)2). Synergy of WG with FA improved the amorphous content of the binder, as bredigite (CaMg(SiO_4_)_4_), hedenbergite (CaFeSi_2_O_6_), and ferrite phases were found to be present in C_80_FA_20_WG_0_. These phases were found absent in C_80_FA_10_WG_10_. Both binary (C_80_FA_20_G_0_) and ternary blended binders (C_80_FA_10_G_10_) have muscovite ((KF)_2_(Al_2_O_3_)_3_(SiO_2_)_4_) and biotite (K(Mg,Fe)_3_AlSi_3_O_10_(F,OH)_2_) as the main crystalline phases. These phases were absent in the OPC system (C_100_FA_0_G_0_), which suggests that the OPC system as a crystalline phase of tobermorite (C-S-H) became amorphous in C_80_FA_10_G_10_ and C_80_FA_20_WG_0_ systems owing to the formation of C-A-S-H.

### 3.6. Bond Characteristics of Binary (FA-OPC) and Ternary Blended (WG/FAP/OPC) Binders

**Figure 11:**
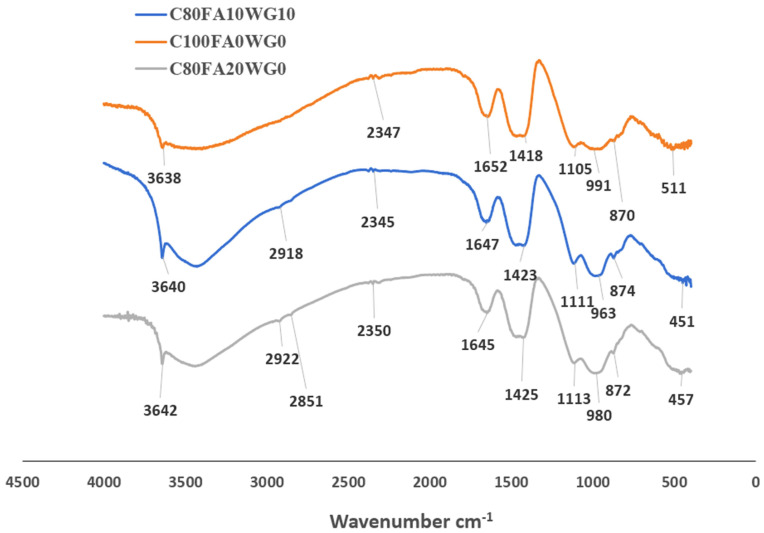
FTIR spectra of hydrated cement (C_100_FA_0_WG_0_—top), glass–fly ash–cement (C_80_F_10_WG_10_—middle), and fly ash blended cement (C_80_FA_20_WG_0_—bottom) paste.

Figure 11 indicates that C_80_FA_10_WG_10_, C_80_FA_20_WG_0_, and pure OPC binder (C_100_FA_0_WG_0_) have different bond characteristics. For instance, the bending vibration of Si-O-Si(Al) was noted at wavenumbers of 511, 451, and 457 cm^−1^, with the highest vibration in C_100_FA_0_WG_0_. The lowest vibrations are found in the FA-based binary system (C_80_FA_20_WG_0_). Yusuf assigned the bending vibration of tetrahedral silicate (Si-O-Si(Al)) at 400–520 cm^−1^ due to the presence of C-S-H, tobermorite, and unreacted alite/belite in the OPC binder. Moreover, there were more Al-O stretching vibrations in C_80_FA_20_G_0_ than in C_80_FA_10_G_10_ due to the presence of Al-O-Si bond vibration. This indicates that the formation of the (C-A-S-H) product is present in FA-based binder (C_80_FA_20_WG_0_). The presence of FA in both C_80_F_10_WG_10_ and C_80_FA_20_WG_0_ is the main reason why Al-O-Si vibrations in both systems are very close in value (451 and 457 cm^−1^). This also explains why the FA-OPC systems (C_80_FA_20_WG_0_) become less amorphous compared to the WG-FA-OPC system (C80FA10G10), as indicated in Figure 10 (XRD results).

Furthermore, the asymmetric stretching of Si-O-Al vibration is 870–874 cm^−1^, with the highest vibration (874 cm^−1^) observed in the more heterogenous ternary blended paste (C_80_FA_10_WG_10_), followed by the less heterogenous FA-OPC (C_80_FA_20_WG_0_) system (872 cm^−1^), while the lowest value was recorded in the OPC system (C_100_FA_0_G_0_) (870 cm^−1^). There are also asymmetric Si-O-Si bond vibrations within 900–1200 cm^−1^ [42] with specific values of 991 cm^−1^ in C_100_FA_0_G_0_, 980 cm^−1^ in OPC-FA binders (C_80_FA_20_WG_0_), and 963 cm^−1^ in the ternary binder (C_80_FA_10_WG_10_). This is due to high Si-O-Si bonds defining tobermorite with the consequent increase in the compressive strength, as presented in Figure 6. The reduction in the strength in the ternary system compared to the OPC system emphasized the replacement of tobermorite (CSH) with C-(A)-S-H.

Moreover, hydrogen bonding (H-OH) vibrations (2918 and 2922 cm^−1^) defined the blended binders (C_80_FA_20_WG_0_ and C_80_FA_10_WG_10_), but this band is absent in OPC system (C_100_FA_0_WG_0_). This could be due to the effect of dilution of the nucleation site, which induces the formation of Al(OH)_3_ during the hydration process at the expense of the formation of portlandite in a blended system (ternary or binary). Adsorbed water vibration is less in C_80_FA_10_G_10_ (1645 cm^−1^) compared to C_80_FA_20_G_0_ (1647 cm^−1^). These adsorbed water molecules could be found within the capillary pores, while the highest value was found in the OPC system compared to other systems. The carbonation process that led to the formation of calcite (O-C-O bonds) was noted at the bands of 1423 and 1425 cm^−1^. The presence of calcite in the XRD shown in Figure 11 indicates that the carbonation processes happening in the three systems are affected by the presence of portlandite. The presence of portlandite could react with atmospheric carbon dioxide to form calcite. All three samples were susceptible to environmental carbonation (O-C-O in CO_3_^2−^) at different wavenumbers ranging from 2345 to 2350 cm^−1^ [43]. The least vibration value was recorded in C_80_FA_10_WG_10_, while the highest was observed in the FA-OPC system (C_100_FA_0_G_10_) due to a difference in their structural parking density. A summary of the identified bands in all the developed concrete mixes is presented in Table 6. However, the significance of these bands in terms of durability performance will need further investigation.

## 4. Conclusions

This study investigates the performance of ternary blended concrete made from ordinary Portland cement (OPC), fly ash (FA), and waste glass (WG) in terms of consistency, absorption, compressive strength, product phases, bonds, and microstructural characteristics, such that WG/(WG + FA + OPC) and FA/(WG + FA + OPC) varied from 0% to 20% at 80% OPC (C_80_FA_20-x_WG_x_) by the weight of the total binders. The following are the main conclusions:

- The impact of WG on FA-blended concrete in affecting the workability performance of the ternary blended concrete could vary depending on the WG/FA ratios in the mixture.
- When WG preponderates FA in the ternary blending, porosity and absorption improved due to differences in the particle size and oxide composition.
- Keeping FA and WG compositions within 20% will produce a structural concrete whose optimum value was 48 MPa at WG and FA contents of 15% and 5%, respectively.
- C_80_FA_10_WG_10_ had better morphological characteristics and microstructural density than C_80_FA_20_WG_0_ due to pore-filling effect.
- Energy-dispersive spectroscopy (EDS) indicates that C_80_FA_10_WG_10_ has higher Si/Al, Si/Ca, and Ca/Al ratios than C_80_FA_20_G_0_.
- Fourier transform infrared spectroscopy (FTIR) reveals that the compressive strength is directly related to Si-O asymmetric stretching vibrations. C_100_FA_0_WG_0_ has the highest frequencies of Si-O-Al bending vibration at wavenumber 511 cm^−1^ and capillary adsorbed water (H-O-H) asymmetric stretching vibration at 1652 cm^−1^ in comparison with binary (C_80_FA_20_WG_0_) and ternary (C_80_FA_10_WG_10_) blended systems.
- An X-ray diffractogram (XRD) indicates that WG contributed to the amorphous content of the ternary binder due to the disappearance of the hedenbergite phase (CaFeSi_2_O_6_) in C_80_FA_10_G_10_, which was found in C_80_FA_20_G_0_.
- There was a phase transformation when WG and FA were incorporated into the OPC system due to the disappearance of the tobermorite (C-S-H) phase and the formation of amorphous C-A-S-H.
- Finally, the use of these solid-waste additives (WG and FA) promotes a reduction in greenhouse gas (CO_2_) due to the partial replacement of OPC, thereby enhancing the reduction in landfills, valorization of solid waste, and economic concrete production.

## Figures and Tables

**Table 1:** Percentage oxide composition of the raw materials.

Oxide Composition	Glass	Fly Ash	OPC
SiO_2_	68.10	60.34	19.01
Al_2_O_3_	0.90	28.11	4.68
Fe_2_O_3_	0.60	3.71	3.20
CaO	14.50	1.34	66.89
MgO	1.80	-	0.81
Na_2_O	12.20	0.55	0.09
TiO_2_	0.00	-	0.22
K_2_O	0.80	1.00	1.17
P_2_O_5_	-	-	0.08
SO_3_	0.40	0.80	3.66
MnO_2_	-	-	0.19
SiO_2_ + Al_2_O_3_ + Fe_2_O_3_	69.60	92.16	26.89
SG	2.48	2.38	3.14
LOI (%)	0.80	0.50	2.80
Surface area (m^2^/g)	0.223	420	0.33
D_10_ (microns)	40	6	-
D_50_ (microns)	160	27	-
D_90_ (microns)	300	120	-

**Table 2:** Coarse aggregate distribution [36].

Coarse Aggregate Size (mm)	Percentage Composition
10	30
12	20
14	30
20	20

**Table 3:** Specific gravities of all materials.

Materials	Specific Gravity Values
Cement	3.14
Glass	2.48
Fly ash	2.38
Sand	2.71
Coarse	2.54

**Table 4:** Mix design for 1 m^3^ ternary blended (OPC+FA+WG) concrete.

Mixes	Fly Ash (%FAs)	Waste Glass (%WG)	OPC (%)	w/b Ratio	OPC (kg/m^3^)	Fly Ash (kg/m^3^)	WG (kg/m^3^)	Fine Agg. (kg/m^3^)	Coarse Agg. (kg/m^3^)	Water SSD	SP	Total Density
C_100_FA_0_WG_0_	0	0	100	0.4	350	0.0	0.0	753	1120	175	1.80	2400
C_80_FA_20_WG_0_	20	0	80	0.4	280	70.0	0.0	760	1120	168	1.80	2400
C_80_FA_15_ WG_5_	15	5	80	0.4	280	52.5	17.5	760	1120	168	1.80	2400
C_80_FA_10_ WG_10_	10	10	80	0.4	280	35.0	35.0	760	1120	168	1.80	2400
C_80_FA_5_WG_15_	5	15	80	0.4	280	17.5	52.5	760	1120	168	1.80	2400
C_80_FA_0_WG_20_	0	20	80	0.4	280	0.0	70	760	1120	168	1.80	2400

**Table 5:** EDS elemental composition of the binders.

Elemental Ratio	Fly Ash–OPC Paste C_80_FA_20_WG_0_	Fly Ash–Glass–OPC Paste C_80_FA_10_WG_10_
Ca/Si	3.82	3.39
Ca/Al	11.46	12.52
Si/Al	3.00	3.69

**Table 6:** FTIR bands of the developed binder.

S/N	C_80_FA_10_WG_10_	C_100_FA_0_WG_0_	C_80_FA_20_WG_0_	Functional Group Assignment
1	3640	3638	3642	O–H stretching (hydroxyl groups, adsorbed water)
2	2918	-	2922	C–H stretching (aliphatic compounds)
3	-	-	2851	C–H symmetric stretching
4	2345	2347	2350	CO_2_ asymmetric stretching (atmospheric CO_2_)
5	1647	1652	1645	H–O–H bending (water)
6	1423	1418	1425	C–O stretching/carbonate group (CO_3_^2−^)
7	1111	1105	1113	Si–O–Si asymmetric stretching
8	963	991	980	Al–O or Si–O vibrations (aluminosilicates)
9	874	870	872	C–O bending (carbonates)
10	451	511	457	Si–O–Si bending vibrations

## Data Availability

The original contributions presented in this study are included in the article. Further inquiries can be directed to the corresponding author.
